# Comparison of the Antianhedonic Effects of Repeated-dose Intravenous Ketamine in Older and Younger Adults with Major Depressive Episode

**DOI:** 10.2174/1570159X23666240923112548

**Published:** 2024-09-23

**Authors:** Wei Zheng, Limei Gu, Jianqiang Tan, Yanling Zhou, Chengyu Wang, Xiaofeng Lan, Bin Zhang, Zezhi Li, Yuping Ning

**Affiliations:** 1 The Affiliated Brain Hospital of Guangzhou Medical University, Guangzhou, China;; 2 The First School of Clinical Medicine, Southern Medical University, Guangzhou, Guangdong, China

**Keywords:** Ketamine, depression, older adults, younger adults, anhedonia, major depressive disorder

## Abstract

**Objectives:**

Growing evidence suggests that repeated-dose intravenous ketamine in patients with depression had rapid antianhedonic effects. However, a comparison of the antianhedonic effects of repeated-dose intravenous ketamine between younger adults and older depressed patients has not been examined.

**Methods:**

To the best of my knowledge, this study with a total of 135 patients with major depressive episodes (MDE) is the first to compare the antianhedonic effects between younger adult (n = 116) and older (n = 19) depressed patients receiving six ketamine infusions (0.5 mg/kg over 40 min). Montgomery-Åsberg Depression Rating Scale (MADRS) was applied in this study to evaluate the clinical symptoms, and MADRS anhedonia item scoring was used to evaluate anhedonia symptoms.

**Results:**

Patients received six open-label intravenous infusions of ketamine for 12 days. MADRS anhedonia subscale scores decreased in both younger (3.3, 95% CI = 2.5-4.1, *p <* 0.05) and older (2.8, 95% CI = 1.1-4.6, *p <* 0.05) MDE patients at 4h after the first infusion compared to baseline scores and the reduction was maintained over the subsequent infusion period in both groups (all *Ps* < 0.05). Younger MDE patients had lower MADRS anhedonia subscale scores on day 26 compared with older patients (*P* = 0.02). Compared with younger adult MDE patients, older patients had a lower antianhedonic response (51.7% [95% CI = 42.5%-61.0%] *versus* 31.6% [95% CI = 8.6%-54.6%)] and remission (24.1% [95% CI = 16.2%-32.0%] *versus* 0%).

**Conclusion:**

This study indicates that repeated-dose intravenous ketamine administration induces rapid and robust antianhedonic effects in older MDE patients. However, older MDE patients displayed less response to ketamine than younger adult MDE patients.

**Registration Number:**

ChicCTR-OOC-17012239.

## 1 INTRODUCTION

Depression is a common but serious psychiatric disorder affecting an estimated 7% of the elderly population [1]. Late-life depression (LLD) has more complications and can co-occur with other more severe medical illnesses than early-onset or adult depression [2, 3]. For example, LLD has a poorer long-term prognosis, a higher recurrence rate, and a greater likelihood of medical morbidities, psychotic symptoms, and neurocognitive impairment when compared with early-onset or adult depression [4-6]. Depression occurring in older patients appears to be undetected or inadequately treated [7].

Accumulating evidence suggests that antidepressants and electroconvulsive therapy (ECT) are effective, relatively safe and well-tolerated in treating older patients suffering from major depressive disorder (MDD) [8]. For example, a recent systematic review reported that ECT is an effective treatment in improving depressive symptoms of older patients suffering from MDD [6]. In addition, numerous studies have investigated the difference in response to antidepressants between older and younger adult depressed patients, but the findings are inconsistent. In general, older depressed patients exhibited lower rates of response and remission [9] and increased side effects induced by antidepressant usage [10]. However, several studies reported that antidepressants are equally effective for acute treatment in either older or younger adult depressed patients [11, 12].

Core symptoms of depression (anhedonia and/or low mood) are likely universal [13, 14]. However, age groups differ in their experience of the condition [15]. As one of the core symptoms of depression, anhedonia occurs in 37% of individuals experiencing major depressive episodes (MDE) [16]. Compared to younger adults, older depressed adults suffer from more severe anhedonia symptoms [17]. Agomelatine [18] or sertraline [19] was safe and effective in treating anhedonia. Anhedonia is an underexplored condition in neuromodulation trials [20]. Recently, several studies found that a single sub-anesthetic dose of ketamine [21, 22] and repeated ketamine [23] or esketamine [24] administration in patients suffering from depression had rapid antianhedonic effects. For example, a significant reduction in anhedonia severity as measured by the Montgomery-Åsberg Depression Rating Scale (MADRS) [25, 26] five-item anhedonia factor scoring was observed after six ketamine infusions [23]. Notably, the antianhedonic effects of a single sub-anesthetic dose (0.5 mg/kg) of ketamine administration [21] and repeated-dose intravenous ketamine administration (0.5 mg/kg over 40 min) [23] were independent on the reduction of other depressive symptoms. Interestingly, the antianhedonic effects of esketamine (0.5-1 mg/kg) [24] and ketamine (0.5 mg/kg over 40 min) [23] did not differ between subjects suffering from MDD or bipolar depression (BD). A recent study reported that younger age was characterized by a faster antidepressant outcome in participants suffering from treatment-refractory depression (TRD) who were treated with six ketamine infusions (0.5 mg/kg) [27]. Given that older depressed patients are associated with worse treatment outcomes than traditional antidepressants when compared to younger depressed patients, the role of ketamine at a sub-anesthetic dose in treating older subjects with depression should be further investigated. Furthermore, the comparative effectiveness of repeated administration of subanaesthetic ketamine (0.5 mg/kg over 40 min) in ameliorating anhedonia between older and younger adult patients with depression has not been investigated.

Thus, the current study aims to investigate the antianhedonic response and remission to repeated administration of subanaesthetic ketamine in older depressed patients and subsequently compare the antianhedonic response and remission to repeated administration of subanaesthetic ketamine between older and younger adult patients with depression.

## 2 METHODS

### 2.1 Participants

This was an open-label, real-world clinical trial (registration number: ChicCTR-OOC-17012239) of ketamine as an adjunctive treatment for subjects who were diagnosed with suicidal ideation and/or TRD [23, 28-30]. The ethics committee at the Affiliated Brain Hospital of Guangzhou Medical University approved the trial protocol (Ethical Application Ref: 2016030). This project was initiated in November 2016 and is ongoing. Written informed consent was obtained from all study participants.

Eligibility criteria were as follows: (1) willingness to provide written informed consent; (2) one hundred and thirty-five participants (ages 18-65, 68 male) who met the DSM-5 criteria for either unipolar or bipolar depression without psychotic symptoms confirmed by two psychiatrists were recruited; (3) all subjects were suffering from an MDE of at least moderate severity (the 17-item Hamilton Rating Scale for Depression [31] score ≥ 17) at the time of screening; (4) participants failed to respond to at least 2 different antidepressant agents [28] or were experiencing suicidal ideation defined by Beck Scale for Suicide Ideation (SSI)-part I ≥ 2 [29]. The exclusion criteria of all recruited subjects included: (1) any other serious mental disorder as defined by DSM-5 criteria, such as schizophrenia; (2) any unstable medical illness; (3) a positive urine pregnancy test if female; (4) a positive urine toxicology screen; (5) lifetime history of neurological diseases such as dementia.

In this study, patients were divided into older (≥ 50 years) and younger adult (< 50 years, ≥ 18 years) groups as recommended previously [32, 33]. In China, the age of ≥ 50 years has frequently been used in numerous clinical studies as the cutoff value for “older adults” [32, 33].

### 2.2 Measures

A self-designed questionnaire including sex, age, height, weight, education level, and marital status was collected through face-to-face interviews. MADRS anhedonia item scoring was used to evaluate anhedonia symptoms as recommended previously [34, 35], and it was evaluated at baseline, at four hours and one day after each infusion, and at two weeks after the completion of ketamine infusions (26 d).

### 2.3 Ketamine Infusions

As recommended previously [36, 37], participants received a sub-anaesthetic dose of ketamine (0.5 mg/kg) diluted in saline (40 ml), which was administered over 40 min *via* IV intravenous pump following an overnight fast. Enrolled participants received open-label repeated administration of subanaesthetic ketamine for 12 days (Monday-Wednesday-Friday). During each injection, the haemodynamic and clinical status of each participant was monitored. During this study, participants were allowed to remain on their psychiatric drugs, such as antidepressants and antipsychotics. Current psychotropic drugs had to be stable for more than four weeks, and the same dose was maintained during this study.

### 2.4 Antianhedonic Response and Remission

On day 13 (1 d after the 6^th^ infusion), a reduction of ≥ 50% and ≥ 75% on the MADRS anhedonia subscale scores was defined as antianhedonic response and remission, respectively [23]. Repeated assessments for the MADRS maintained an interclass correlation coefficient (ICC) > 0.90.

### 2.5 Statistical Analysis

An intent-to-treat (ITT) analysis of anhedonia symptoms as measured by MADRS was carried out for all individuals suffering from depression who had a baseline assessment and at least another assessment [38]. Data were analyzed using SPSS 24.0 statistical software. Comparisons of demographic data and baseline ratings between older and younger adult patients with depression were conducted by using the Student’s t-test and/or Mann-Whitney U test and the Chi-square and/or Fisher's exact tests for continuous and categorical data, respectively, as appropriate. The rate of antianhedonic response and remission between older and younger adult patients with depression groups were compared after adjusting for significant confounding variables. The severity of anhedonia symptoms was compared at each assessment time point between younger and older depressed patients using linear mixed models. Changes between two-time points for anhedonia symptoms were compared using paired t-tests, as appropriate. Significance was set to *p* < 0.05.

## 3 RESULTS

### 3.1 The Demographic and Clinical Characteristics of Participants

**Fig. (1):**
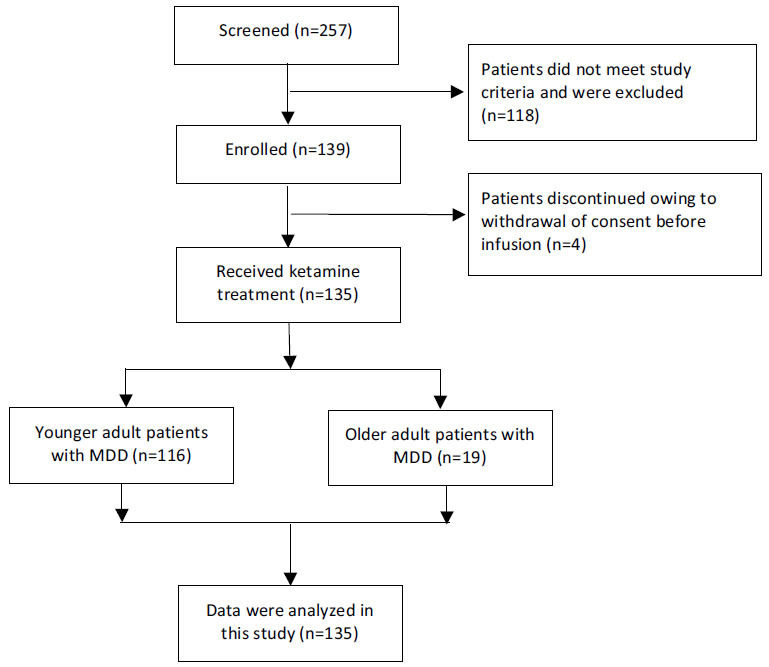
Flow chart of the patient inclusion process.

The full details of the demographic and clinical characteristics were reported previously [39]. A total of 135 MDE patients were recruited, including patients with MDD (n=103) and BD (n=32) (Fig. **1**). Sixty-eight (50.4%) males were recruited in this study. The mean age and duration of illness were 34.8 ± 11.7 years and 102.0 ± 91.8 months, respectively. The mean baseline HAMD-17 scores, MADRS scores, and MADRS anhedonia subscale scores were 23.8 ± 5.1, 32.8 ± 7.9, and 20.4 ± 4.7, respectively.

### 3.2 Demographic and Clinical Characteristics of Older and Younger Adult MDE Patients

According to age, 116 patients were determined as younger adult patients (n=116), while 19 were determined as older patients. The full details of the demographic and clinical characteristics of older *versus* younger adult MDE patients receiving six ketamine infusions were reported previously [39]. Older MDE patients were also less likely to be married and have a higher age of onset (all *p <* 0.05). No significant differences between the two groups were found in terms of baseline MADRS anhedonia subscale scores, baseline MADRS scores, and baseline HAMD-17 scores (all *p* > 0.05, Table **1**).

### 3.3 Difference in Antianhedonic Score between Older and Younger adult MDE Patients after Ketamine Infusions

After a 13-day follow-up, linear mixed models showed that there was a significant time effect (F = 37.7, *p <* 0.001) and time * group interaction effect (F = 3.1, *p <* 0.001) measured by MADRS anhedonia subscale scores, but no group main effects were detected (*P* = 0.44). As shown in Fig. (**2**), significant reductions in MADRS anhedonia subscale scores in either younger (3.3, 95% CI: 2.5-4.1; t = 8.7, *p <* 0.001) or older (2.8, 95% CI: 1.1-4.6; t = 3.4, *P* = 0.003) MDE patients groups were found at 4h after the first infusion compared to baseline scores and maintained over the subsequent infusion period in both groups (all *Ps* < 0.05). However, with regard to the difference in antianhedonic effects of ketamine, a significant difference between the two age groups was found in the ketamine’s antianhedonic effects only on day 26 (*P* = 0.02, Fig. **1**).

### 3.4 Difference in Antianhedonic Response and Remission Rate between Older and Younger Adult MDE Patients after Ketamine Infusions

On day 13 (1 d after the 6^th^ infusion), 66 patients (48.9%; 95 confidence interval [CI] = 40.3%-57.4%) and 28 patients (20.7%; 95 CI=13.8%-27.7%) were identified as having antianhedonic response and remission, respectively.

Older MDE patients had a trend to display a lower antianhedonic response rate (31.6% [95% CI = 8.6%-54.6%] *versus* 51.7% [95% CI = 42.5%-61.0%], χ^2^=2.6, *P* = 0.10), compared with younger adult MDE patients. Furthermore, Older MDE patients were more likely to have a lower remission rate (0% *versus* 24.1% [95% CI = 16.2%-32.0%], χ^2^=9.1, *P* = 0.003) even after adjusting for significant confounding variables (Table **1**).

## 4 DISCUSSION

To the best of our knowledge, this is the first study in a real-world setting to examine and compare the antianhedonic effectiveness of intravenous ketamine infusions among older compared with younger adult MDE patients. The main findings were: (1) multi-infusion ketamine treatment showed rapid and robust antianhedonic effects in older MDE patients, and (2) older MDE patients were less responsive to ketamine’s antianhedonic effects compared with younger adult MDE patients and reported lower response and remission rates. In addition to the rapid antidepressant and antisuicidal effects of ketamine [28, 29, 37, 40, 41], the rapid antianhedonic effects of ketamine have been reported in MDD and BD [21, 22]. Importantly, the antianhedonic effects of ketamine at subanesthetic doses occur independently on the reduction of depressive symptoms [21, 23]. A naturalistic multicentric study found that esketamine nasal spray was safe and effective in treating treatment-resistant bipolar depression (TRBD) [42]. Importantly, no significant differences were found in terms of response or remission rates between patients suffering from TRBD and TRD receiving esketamine nasal spray [42]. Therefore, ketamine/esketamine could be considered a valuable tool to stabilize subjects with mixed symptoms or bipolar spectrum [43]. Furthermore, baseline plasma brain-derived neurotrophic factor (BDNF) levels are correlated with the antianhedonic effects of multi-infusion ketamine treatment in MDD [44].

The reduction of anhedonia symptoms in this study was observed within 4 h after the first ketamine infusion, and the effect was maintained following the subsequent ketamine infusions. Following six ketamine infusions, 31.6% of older patients with depression met the antianhedonic response criteria. However, none of the older MDE patients reached the antianhedonic remission criteria. The antidepressant effects of ketamine or esketamine on older MDE patients have been examined but with inconsistent findings [45-47]. For example, several studies reported that subanaesthetic ketamine administration was effective and safe in ameliorating depressive symptoms in older MDE patients (≥ 55 years) [46, 47]. However, Ochs-Ross *et al.* reported that flexibly-dosed esketamine did not significantly alleviate depressive symptoms in older subjects experiencing TRD (≥ 65 years) compared to the control group [45]. Mashour *et al.* showed that intraoperative administration of subanaesthetic ketamine also failed to alleviate depressive symptoms in older adults [48]. Therefore, the ketamine’s antidepressant and antianhedonic effects on subjects with older MDE should be further studied.

A recent study focusing on child psychiatric patients found that anhedonia symptoms were more frequent in the older age group [49]. Similarly, age appeared to affect the trajectory of the antidepressant effects of repeated intravenous ketamine infusions [27]. In this study, older MDE patients (≥ 50 years) were more likely to have a lower antianhedonic response and remission when compared with younger adult MDE patients (< 50 years). In general, older patients with depression were typically associated with lower antidepressant response and remission rates when compared with younger patients with depression [9]. However, a recent study found a generally comparable improvement in depression between older and younger adult patients suffering from TRD who received esketamine nasal spray and oral antidepressants [50]. The highest antianhedonic effects occurred at the fourth ketamine infusion in older MDE patients, but the reason remained unclear.

Our study had several important limitations. First, the two patient populations have totally different sizes (116 in younger patients *versus* 19 in older patients), potentially explaining the negative findings for antianhedonic response between the older and younger patient groups. Second, similar to other studies [50], the lack of control data would inevitably be impacted by subjective evaluation. Third, the measurement of anhedonia symptoms was performed based on the analysis of the MADRS anhedonia subscale instead of using a specific scale such as the Snaith-Hamilton Pleasure Scale (SHAPS) [51] or the Dimensional Anhedonia Rating Scale (DARS) [52] for anhedonia symptoms in this study. Fourth, the study did not distinguish between participants diagnosed with unipolar and bipolar depression, even though some studies have found no difference on the antianhedonic effects of esketamine [21] and ketamine [20] between subjects experiencing MDD and BD. However, the use of the MADRS anhedonia subscale to evaluate anhedonia symptoms has been employed with success in recently published studies [23, 34, 53]. Finally, the toxic effects of ketamine on the genitourinary system were first reported in 2007 [54]. However, ketamine-induced genitourinary side effects were not collected in this study.

## CONCLUSION

In conclusion, our study indicates that repeated-dose intravenous ketamine administration produces rapid and robust antianhedonic effects in older MDE patients. Older MDE patients show lower antianhedonic response to multi-infusion ketamine treatment when compared with younger adult MDE patients.

## Figures and Tables

**Fig. (2):**
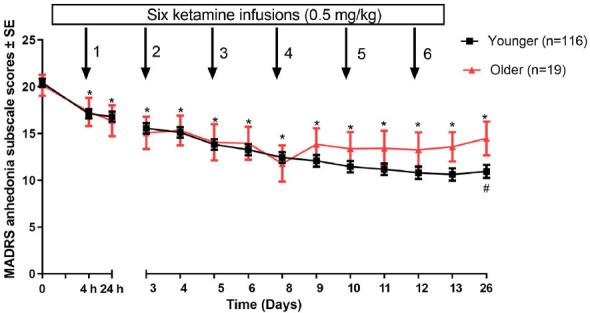
Change in anhedonia symptoms of older and younger MDE patients following six ketamine infusions. *There was a significant difference at a given time point when compared to baseline (*P* < 0.05). ^#^There was a significant difference at a given time point between younger (< 50 years) and older (≥ 50 years) MDE patients groups (*P* < 0.05). **Abbreviations:** MADRS = Montgomery-Åsberg Depression Rating Scale; SE = standard error.

**Table 1:** Demographic and clinical characteristics of sample.

**Variables**	**Total Sample ** **(n=135)**		**Younger Group (n=116)**		**Older Group ** **(n=19)**		**Statistics**		
	**N**	**%**	**N**	**%**	**N**	**%**	**χ^2^**	**df**	***p***
Antianhedonic response rate	66	48.9	60	51.7	6	31.6	2.6	1	0.10
Antianhedonic remission rate	28	20.7	28	24.1	0	0	9.1	1	**0.003**
	Mean	SD	Mean	SD	Mean	SD	T/Z	df	*p*
Baseline HAMD-17 scores	23.8	5.1	23.9	5.1	22.8	5.0	0.8	133	0.40
Baseline MADRS scores	32.8	7.9	32.9	7.7	31.9	8.9	0.5	133	0.62
Baseline MADRS anhedonia subscale scores	20.4	4.7	20.4	4.7	20.2	4.9	0.2	133	0.82

**Note**: ^a^Fisher’s exact test. ^b^Mann-Whitney U test. Bolded values are *p* < 0.05. **Abbreviations:** HAMD-17=17-item Hamilton Depression Rating Scale; MADRS=Montgomery-Åsberg Depression Rating Scale.

## Data Availability

The data that support the findings of this study are available from the corresponding author Yuping Ning upon reasonable request.

## LIST OF ABBREVIATIONS

LLD: Late-life Depression
ECT: Electroconvulsive Therapy
MDD: Major Depressive Disorder
MADRS: Montgomery-Åsberg Depression Rating Scale
BD: Bipolar Depression
TRD: Treatment-refractory Depression
TRBD: Treatment-resistant Bipolar Depression
BDNF: Brain-derived Neurotrophic Factor

## STANDARDS OF REPORTING

CONSORT Checklist and methodology were followed.
